# Food insecurity and kidney disease: a systematic review

**DOI:** 10.1007/s11255-023-03777-w

**Published:** 2023-09-08

**Authors:** Francesca Ferrara, Rossella Siligato, Alessio Di Maria, Laura Scichilone, Emanuele Di Simone, Marta Bondanelli, Alda Storari, Alfredo De Giorgi, Marco Di Muzio, Fabio Fabbian

**Affiliations:** 1 Renal Unit, University Hospital of Ferrara, 44124 Ferrara, Italy; 2 Department of Clinical and Molecular Medicine, Sapienza University of Rome, 00185 Rome , Italy; 3 Department of Medical Sciences, University of Ferrara, Via Luigi Borsari 46, 44121 Ferrara, Italy; 4 Clinica Medica Unit, University Hospital of Ferrara, 44124 Ferrara, Italy

**Keywords:** Food insecurity, Chronic kidney disease, Systematic review

## Abstract

**Background:**

The risk of developing and worsening chronic kidney disease (CKD) is associated with unhealthy dietary patterns. Food insecurity is defined by a limited or uncertain availability of nutritionally adequate and safe food; it is also associated with several chronic medical conditions. The aim of this systematic review is to investigate the current knowledge about the relationship between food insecurity and renal disease.

**Methods:**

We selected the pertinent publications by searching on the PubMed, Scopus, and the Web of Science databases, without any temporal limitations being imposed. The searching and selecting processes were carried out through pinpointed inclusion and exclusion criteria and in accordance with the Prisma statement.

**Results:**

Out of the 26,548 items that were first identified, only 9 studies were included in the systemic review. Eight out of the nine investigations were conducted in the US, and one was conducted in Iran. The studies evaluated the relationship between food insecurity and (i) kidney disease in children, (ii) kidney stones, (iii) CKD, (iv) cardiorenal syndrome, and (v) end stage renal disease (ESRD). In total, the different research groups enrolled 49,533 subjects, and food insecurity was reported to be a risk factor for hospitalization, kidney stones, CKD, ESRD, and mortality.

**Conclusions:**

The relationship between food insecurity and renal disease has been underestimated. Food insecurity is a serious risk factor for health problems in both wealthy and poor populations; however, the true prevalence of the condition is unknown. Healthcare professionals need to take action to prevent the dramatic effect of food insecurity on CKD and on other chronic clinical conditions.

**Supplementary Information:**

The online version contains supplementary material available at 10.1007/s11255-023-03777-w.

## Introduction

A recent paper has evaluated the articles, published between 2011 and 2020, that dealt with the impact of modifiable lifestyle factors on the incidence and/or progression of chronic kidney disease (CKD). The authors performed a bibliometric analysis and found that diet, obesity, and physical activity were the most important risk factors that were studied by different research groups [1]. There is already a significant association between unhealthy dietary patterns and an increased risk of developing or worsening CKD [2]. Moreover, socially disadvantaged persons experience a disproportionate burden of kidney disease worldwide [3]. The right to an adequate standard of living includes access to food and should be one of the universal human rights [4]. The present living conditions are influenced by global crises with impacts on social epidemiology and public health. A lack of reliable approaches to the relevant, safe, and nourishing foods that are required for a healthy life defines food insecurity; food insecurity means that people are not provided with enough food for an active and healthy life [5]. The US Department of Agriculture (USDA) defines food insecurity as “limited or uncertain availability of nutritionally adequate and safe foods, or limited or uncertain ability to acquire acceptable foods in socially acceptable ways” [6]. Such a problem is strongly connected to income; however, food insecurity is detected in both developed and developing countries. In wealthy nations food insecurity has been related to overnutrition, meaning an overconsumption of nutrients causing obesity and cardiometabolic diseases. In contrast, food insecurity in low-income nations causes malnutrition, meaning undernutrition and starvation [7]. The majority of data about the health impact of food insecurity in western societies come from the United States (US). In the US, the prevalence of food insecurity is high in low income non-Hispanic, Black, and Hispanic households [8]. In addition, it is associated with several chronic medical conditions [9]; psychological distress, such as depression, stress, and anxiety [10]; cost-related medication and healthcare underuse [11]; and badly managed health services [12].

Chronic kidney disease (CKD) is a considerable global health challenge, it is a major risk factor for cardiovascular disease, death, and end-stage renal disease. The age-standardized global prevalence of CKD stages 1–5, in adults aged 20 and older, is 8.6% in men and 9.6% in women in high-income countries, and 10.6% in men and 12.5% in women in low- and middle-income countries [13].

The prevalence of CKD has been reported to be associated with lower incomes, as well as with lower education [14]. Rapid urbanization in low income countries could be associated with a growing number of people with diabetes and hypertension, which are the leading causes of CKD.

Food insecurity is a condition that healthcare professionals encounter either directly or indirectly, and it is associated with both overnutrition and undernutrition [15]. Food insecure people suffer more chronic and mental health problems, as well as difficulty in purchasing medication [16]. This is because the consumption of healthy food decreases with increasing food insecurity [17]. The social impact of food insecurity is upsetting, food insecurity mothers compromise their own nutritional intake to preserve the adequacy of their children's diets [18]. Food insecurity is associated with diet-sensitive chronic disease, such as hypertension and diabetes [19], probably due to the development of obesity. In fact, food insecurity could promote a susceptibility to weight gain [20]. The aim of this systematic review is to investigate the current knowledge about the relationship between food insecurity and renal diseases.

## Materials and methods

### Literature search

**Fig. 1:**
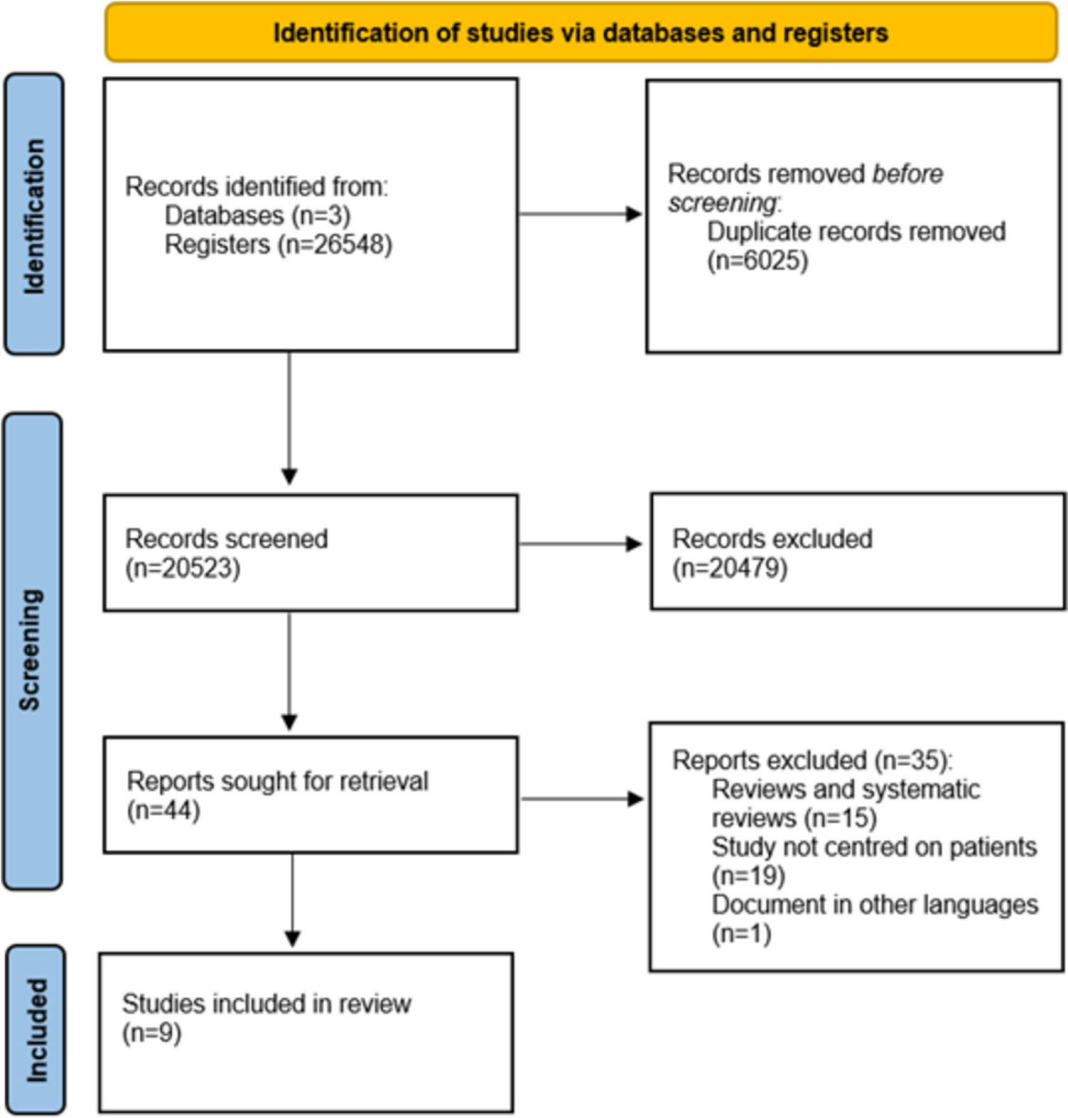
Flow diagram of the search and selection process, based on the PRISMA flowchart

We selected the pertinent publications by searching on PubMed, Scopus, and the Web of Science databases, without any temporal limitations being imposed. The searching strategy is reported in supplementary Table 1, and the selecting process was carried out according to the Prisma statement [21], as shown in Fig. 1. The articles were independently searched by researchers and then discussed if a disagreement occurred.

### Inclusion and exclusion criteria

The inclusion criteria were as follows: (1) studies that quantified food insecurity in people with renal disease; (2) original studies evaluating the relationship between food in-security and the presence or development of renal diseases; and (3) papers published in the English language. We excluded (1) animal studies, (2) editorials, (3) personal opinions, (4) qualitative studies, and (5) review articles.

### Information extraction

The bibliographies of the selected articles were analyzed to detect further data. After reading the full articles, the following information was extracted from the retained final studies: authors, year of publication, country where the study was carried out, study design, population investigated, and main findings. In addition, the full text articles were examined to exclude duplicate entries by the same first or corresponding author, and they were also judged as to whether there were overlaps in content. Then, full text versions of the included articles were evaluated by the authors to compile a table summarizing the main findings about the relationship between food insecurity and renal diseases.

### Evaluation of the quality of selected studies and the assessment of the risk of bias

The evaluation of the quality of selected studies and the assessment of the risk of bias was carried out with JBI appraisal checklist (https://jbi.global/critical-appraisal-tools). JBI is a global organization promoting and supporting evidence-based decisions aiming at improving health outcomes by the use of the best available evidence (https://jbi.global/about-jbi).

## Results

The search strategy is reported in Fig. 1. A total of 26,548 items were first identified, and 6025 duplicates were excluded. During the screening phase, out of the 20,523 articles, 44 full text papers were retrieved and read. Subsequently 35 reports were excluded: 19 items because they were reviews, 14 because they were not evaluating the population’s health, and 1 because it was not written in English. After an accurate selection process, 9 articles [22–30] met the inclusion criteria and were included in the systematic review. We identified 9 studies, the oldest of which was published in 2006 and the newest in 2021. Eight out of the nine investigations were conducted in the US, and one was conducted in Iran. Four studies were cross-sectional studies, and five were observational ones. Two studies from the same research group evaluated children with kidney disease, two studies enrolled patients with kidney stones, three studies evaluated subjects with CKD, and the last two papers analyzed cardiorenal and end-stage renal disease (ESRD) patients, who were treated with hemodialysis. In total, the different research groups enrolled 49,533 subjects, and food insecurity was reported to be a risk factor for hospitalization, for the presence and development of kidney stones, CKD, progression to ESRD, and mortality. The author, year of publication, country where the study was carried out, study design, the population investigated, and main findings are reported in Table 1.

**Table 1:** Author, year of publication, country where the study was carried out, study design, population investigated and the main findings of the studies analyzing the relationship between food insecurity and renal diseases

Author	Year of publication	Country	Study design	Population	Main findings
Children					
Starr [22]	2018	US	Cross-sectional study	118 outpatients children with CKD	35% of children were living in food insecure households
Starr [23]	2019	US	Cross-sectional study	44 children with ESRD	64% were suffering from food insecurity. Children with food insecurity were younger and had higher unplanned hospital or intensive care unit admissions; the prevalence of infections was higher in food insecure children
Kidney stones					
Shafi [24]	2017	Iran	Cross-sectional study	100 patients with calcium oxalate kidney stones and 100 subjects as the control group	68% of patients were suffering from food insecurity (vs. 40% in the control group); food insecurity was associated with a diagnosis of kidney stones
Bayne [25]	2021	US	Observational study	1496 patients with kidney stones of whom 324 were relapsing and had thus underwent surgery	Subjects living in low income census tracts had a higher risk of undergoing re-intervention
Chronic kidney disease					
Terrell [26]	2009	US	Observational study. Data derived from National Health and Nutrition Examination Survey (NHANES) 1999–2004	15,199 people aged 45 years, 63% lived above the poverty income ratio. A total of 10% reported food insecurity, and 17% had kidney disease	17.04% of people with CKD were suffering from food insecurity. 82.59% of respondents with proteinuria reported food insecurity. There was no significant association between food insecurity and CKD nor its control
Crews [27]	2014	US	Observational study. Data form the National Health and Nutrition Examination Survey (NHANES) 2003–2004, 2005–2006, 2007–2008	9,126 individuals aged 46 ± 0.4 years who had a household income < 400% of the Federal Poverty Level	11% were suffering from marginal food insecurity, and 15% from high food insecurity. CKD was associated with food insecurity in patients with diabetes and hypertension; moreover, CKD was associated with food insecurity
			Data from the National Institute of Aging (NIA), Healthy Aging in Neighborhoods of Diversity across Life Span (HANDLS) study	1,239 individuals aged 30–64 years	
Banerjee [28]	2017	US	Observational study. Data derived from National Health and Nutrition Examination Survey (NHANES) 1988–1994	2320 subjects with CKD and 10,448 non-CKD participants with a household income ≤ 400% of the Federal Poverty Level	4.5% of CKD patients were suffering from food insecurity (vs. 5.7% of non-CKD subjects), and this group was more likely to progress to ESRD
Hemodialysis					
Wilson [29]	2006	US	Cross-sectional study	98 hemodialysis patients	16.3% were suffering from food insecurity and they were mainly African American
Cardiorenal syndrome					
Banerjee [30]	2019	US	Observational study. Data derived from National Health and Nutrition Examination Survey (NHANES) 1999–2010	9,245 subjects aged 45 ± 0.29 years earning < 130% of the Federal Poverty Level Guidelines	37.8% were suffering from food insecurity; the risk of all-cause mortality was higher among the individuals with cardiorenal syndrome in terms of suffering from food insecurity

The evaluation of the quality of selected studies and the assessment of the risk of bias is reported in Tables 2 and 3.

**Table 2:** Evaluation of the quality of the studies analysed and the assessment of the risk of bias using JBI critical appraisal checklist for analytical cross sectional studies

	Starr 2018 [22]	Starr 2019 [23]	Shafi 2017 [24]	Wilson 2006 [29]
Were the criteria for inclusion in the sample clearly defined?	Yes	Yes	Yes	Yes
Were the study subjects and the setting described in detail?	Yes	Yes	Yes	Yes
Was the exposure measured in a valid and reliable way?	Yes	Yes	Yes	Yes
Were objective, standard criteria used for measurement of the condition?	Yes	Yes	Yes	Yes
Were confounding factors identified?	No	No	Yes	Yes
Were strategies to deal with confounding factors stated?	No	No	Yes	Yes
Were outcomes measured in a valid a reliable way?	Yes	Yes	Yes	Yes
Was appropriate statistical analysis used?	Yes	Yes	Yes	Yes

**Table 3:** Evaluation of the quality of the studies analysed and the assessment of the risk of bias using JBI critical appraisal checklist for cohort studies

	Bayne 2021 [25]	Terrell 2009 [26]	Crews 2014 [27]	Banerjee 2017 [28]	Banerjee 2019 [28]
Were the two groups similar and recruited from the same population?	Yes	Yes	Yes	Yes	Yes
Were the exposures measured similar to assign people to both exposed and unexposed groups?	Yes	Yes	Yes	Yes	Yes
Was the exposure measured in a valid and reliable way?	Yes	Yes	Yes	Yes	Yes
Were confounding factors identified?	Yes	Yes	Yes	Yes	Yes
Were strategies to deal with confounding factors stated?	Yes	Yes	Yes	Yes	Yes
Were the groups/participants free of the outcome at the start of the study (or at the moment of exposure)?	Yes	Yes	Yes	Yes	Yes
Was the outcomes measured in a valid and reliable way?	Yes	Yes	Yes	Yes	Yes
Was the follow-up time reported and sufficient to be long enough for outcomes to occur?	Yes	Yes	Yes	Yes	Yes
Was follow-up complete, and if not, were the reasons to loss to follow-up described and explored?	Yes	Yes	Yes	Yes	Yes
Were strategies to address incomplete follow-up utilized?	Yes	Yes	Yes	Yes	Yes
Was appropriate statistical analysis used?	Yes	Yes	Yes	Yes	Yes

## Discussion

Our main takeaway is that the worldwide knowledge regarding the relationship between food insecurity and renal disease is underinvestigated. On the other hand, a precise estimation of the prevalence and incidence of food insecurity is also lacking and, up to now, there was no internationally accepted way to detect such a problem. Without this information, it is not possible to precisely understand the relationship between renal diseases and food insecurity that appears to involve underprivileged populations.

Food insecurity could be defined as the absence of sufficient food for every person in a household to live an active and healthy life for a short or long period of time. The measurement of food insecurity should suggest the proportion of people who cannot afford food. Poverty, unemployment, low income, lack of affordable housing, chronic health conditions, lack of access to healthcare, and racial discrimination could all be considered causes of food insecurity. A significant number of people living in the United States struggle to meet their basic needs, thus resulting in adults developing serious health issues and children being unable to grow healthily [31].

Food insecurity is usually evaluated with tools that are able to measure the compromises made in food intake. This is achieved by determining how the act of running out of food or money to buy food, skipping meals, and buying cheaper food relates to poor nutrition. Food insecurity is usually classified into three categories: food security, low food security, and very low food security, and it is based on the score derived from the Household Food Security Scale measure, which is a scale created by the US Department of Agriculture [32]. Our analysis appears to exclude the populations living in the poorest countries, and it also showed that food insecurity was a risk factor for hospitalization, as well as for the presence and development of kidney stones, CKD, progression to ESRD, and mortality. An association between CKD, poor dietary habits, and cardiovascular risk factors [12, 33] has been reported. Furthermore, CKD is strongly associated with cardiovascular diseases [34–36]. We could hypothesize that CKD, food insecurity, and cardiovascular diseases form a vicious circle, resulting in the worsening of clinical conditions. Moreover, CKD is a risk factor for the development of complications in diabetic patients [37, 38], and food insecurity is a risk factor for poor glycemic control [12, 39]. Therefore, an evaluation of the social determinants of health should be considered by physicians, as they are associated with mortality [40].

A global crisis has a strong impact on employment and poverty, and the most vulnerable people are the most concerned by financial changes. The cost of living impacts poor citizens, irrespective of the continent, country, or urban or rural area where they live. Poor people need to adapt and find coping strategies to survive [41]. In Italy, relative household and absolute poverty incidence rate (% of households in poverty) is 10.1% and 7.7%, respectively. In addition, the individual relative and absolute poverty incidence rates (% of persons living in households in poverty) are 13.5% and 9.4%, respectively [42]. In Italy, there is no established public aid service that is dedicated to food, as this assistance is delegated to non-government organizations instead. The prevalence of food insecurity in Europe was determined in a French study that was conducted in the Paris metropolitan area. Authors found that the overall food insecurity prevalence was 6.3%, which was higher in poor households that had numerous members and were receiving welfare [43]. From a worldwide perspective, the Food and Agriculture Organization of the United Nations (FAO) estimates 800–900 million undernourished people, which is considered a gross underestimation of the prevalence of food insecurity [44].

Household food insecurity has also become a serious health problem in high-income countries. In the US, the overall prevalence of food insecurity is 10.5% [45]. However, outside North American countries the problem is largely overlooked [46], as is shown by the results of our study. The Food and Agriculture Organization of the United Nations reported that, during 2020, in Africa 59.6% of the population were food insecure; in Asia, the percentage was 25.8; in Latin America and the Caribbean, it was 40.9; and in Northern America and Europe, it was 8.8 [47]. These data should be related to chronic diseases, especially those that are associated with metabolic derangement.

Diet impacts urine composition and modulates the risk of kidney stones [48]. Increasing poverty increases the calcium in urine. A decreasing level of education increases the calcium in urine, as well as the supersaturation of calcium oxalate and calcium phosphate [49, 50]. Ferraro et al. evaluated the modifiable risk factors for the development of kidney stones, including body mass index, fluid intake, DASH (dietary approaches to stop hypertension) style diets, dietary calcium intake, and sugar sweetened beverage intake. They found that the population attributable fraction ranged from 4.4%, for a higher intake of sugar sweetened beverages, to 26.0%, for a lower fluid intake. The population attributable fraction for all five risk factors combined was 57.0% [51].

A lower socioeconomic status is a frequent predictor of the relationship between food insecurity and chronic diseases; in addition, food insecurity could induce nutritional deficiencies and encourage an intake of cheap, heavily processed foods, which are known to be a risk factor for the development of medical chronic conditions [9]. Moreover, food insecurity could contribute to the development of non-communicable diseases by inducing poor people to choose between healthy food and medications [11]. Unhealthy dietary habits are common among urban individuals living in poverty; furthermore, they are also reported to be associated with CKD [33]. Moreover, impaired access to healthy food is secondary to the geographic and financial barriers that are also associated with CKD [52].

Food insecurity and chronic diseases are associated, and obesity is assumed to be the parameter mediating such an association. However, the other three different mechanisms should be taken into consideration: the economic aspect, the dysregulated eating pattern, and the exposure to environmental toxins [9]. Food insecurity should become increasingly recognized as a significant issue in renal diseases, and our results confirm that there is scant research documenting the impact of food insecurity on disease management and the quality of life for those living with CKD and end-stage renal disease (ESRD). On the other hand, there is also scarce information, from a worldwide perspective, on food access for people living with ESRD. Due to the important effect of diet with respect to renal disease, people living with food insecurity and CKD might experience significant challenges in accessing the food necessary for adhering to dietary guidelines. We believe that clinicians should pay attention to the capacity of patients to adhere to therapeutic dietary guidelines, and that studies in this field are necessary. The prevention of CKD could be targeted by resolving the limited access to healthy food due to geographical or financial obstacles [52].

It is also necessary to underline that different studies reported opposing findings. Ozieh et al. [40] evaluated the data from National Health and Nutrition Examination Surveys (2005–2014), including 1376 adults with diabetes and CKD, and found that food insecurity was not independently associated with mortality. Moreover, a recent meta-analysis investigating the relationship between food insecurity and clinically determined type 2 diabetes mellitus could not detect any significant association [53].

In our opinion, this study is important, because it underlines that food insecurity should be considered in CKD patients. Low income is associated with CKD [14], and food insecurity prevalence is higher in households with a lower income. This systematic review could raise the awareness of food insecurity in CKD subjects who are living in low income households.

### Limitations

An estimation of food insecurity prevalence could be difficult to evaluate due to the different characteristics of the global populations. Methods used for detecting food insecurity, survey methodologies, and the time frames considered could all influence results. It should be considered that food insecurity is identified by self-reported questionnaires and could be biased by social and economic conditions. The surveys could have excluded subjects with very low incomes or those who were less educated. The studies were mainly conducted in the US, and only one was carried out in a non-English speaking country. The latter finding could be due to the national healthcare system organization. In Europe, the majority of healthcare systems are universal. However, to the contrary, the US healthcare system does not provide universal coverage and can be defined as a mixed system, where the publicly financed government ran Medicare and Medicaid health coverage coexists with privately financed (i.e., private health insurance plans) market coverage. Most likely food insecurity is not considered a real problem in areas, where the national healthcare system is universal. Moreover, the distinguishing of food insecurity in children and adults could be conducted incorrectly. As such, the best way, most likely, to investigate the problem is to consider households. All these factors could lead to an underestimation of food insecurity and thus a low representation of the groups of people who are at a higher risk of food insecurity. The papers that we selected mainly analyzed populations that suffered food insecurity related to overnutrition, whilst people undergoing malnutrition were never assessed. Food insecurity is rarely the only problem of low income people, economic constraints could be the reason for different physical, mental and social health problems that are rarely considered together. This is because food insecurity is considered but one of the several social variables that are associated with chronic diseases. Finally, results of the evaluated studies do not distinguish hyper- from hyponutrition, preventing the deep discussion about the strength of the possible associations with food insecurity. All these limitations are due to the fact that the literature appears to be biased toward studies based on North America.

## Conclusions

Different negative health outcomes have been reported in adults who are food insecure, such as obesity and other chronic diseases. Moreover, obesity has higher prevalence in food insecure and reduced frequency, quality, variety, and quantity of consumed foods may have a negative effect on children’s mental health [54]. Non-communicable diseases are also known as chronic diseases, their duration is long and they are the result of a combination of genetic, physiological, environmental and behavioural factors. Poverty is closely linked with non-communicable diseases [55]. CKD is a non-communicable disease frequently caused by diabetes and hypertension [56]. Our findings, although descriptive due to the high heterogeneity of the studies considered, highlight the relationship between food insecurity defined as a household level economic and social condition of limited or uncertain access to adequate food and development of renal disease. We underline that social factors should be considered as risk factors for development of renal disease and interventions are required to limit its prevalence especially in low-income society. Further studies are required for investigating this relationship.

Social and economic factors, such as income, education, employment, community safety, social supports, and healthcare, can affect food insecurity, as they are all significantly tied to health problems. Thus, food insecurity should be investigated as a modifiable risk factor for the development and worsening of long-term organ dysfunctions, such as CKD. Furthermore, coping strategies should be established to ameliorate the health consequences associated with food insecurity and to prevent the dramatic worsening of clinical conditions.

### Supplementary Information

Below is the link to the electronic supplementary material.

Supplementary file1 (DOCX 12 KB)

## Data Availability

The data sets generated and/or analysed during the current study are not publicly available, but are available from the corresponding author on reasonable request.
