# Supplementary material for: Food insecurity and kidney disease: a systematic review
Source: Int Urol Nephrol. 2023 Sep 8;56(3):1035–44. doi: 10.1007/s11255-023-03777-w (PMC10853316; doi:10.1007/s11255-023-03777-w)
Supplement: Supplementary file 1 — Supplementary file1 (DOCX 12 KB) [file 11255_2023_3777_MOESM1_ESM.docx]

Supplementary table 1. Searching strategy aiming at investigating the relationship between food insecurity and renal disease.

("chronic kidney disease"[Title/Abstract] OR "renal insufficiency chron-ic"[Title/Abstract] OR "chronic renal insufficiencies"[Title/Abstract] OR (("renal insuffi-ciency"[MeSH Terms] OR ("Renal"[All Fields] AND "Insufficiency"[All Fields]) OR "renal insufficiency"[All Fields] OR ("Renal"[All Fields] AND "Insufficiencies"[All Fields]) OR "renal insufficien-cies"[All Fields]) AND "Chronic"[Title/Abstract]) OR "chronic renal in-sufficien-cy"[Title/Abstract] OR "kidney insufficiency chronic"[Title/Abstract] OR "chronic kidney insufficiency"[Title/Abstract] OR (("Chronic"[All Fields] OR "chronical"[All Fields] OR "chronically"[All Fields] OR "chronicities"[All Fields] OR "chronicity"[All Fields] OR "chronicization"[All Fields] OR "chronics"[All Fields]) AND "kidney insuffi-cien-cies"[Title/Abstract]) OR (("renal insufficiency"[MeSH Terms] OR ("Renal"[All Fields] AND "Insufficiency"[All Fields]) OR "renal insufficiency"[All Fields] OR ("Kidney"[All Fields] AND "Insufficiencies"[All Fields]) OR "kidney insufficiencies"[All Fields]) AND "Chron-ic"[Title/Abstract]) OR "chronic kidney diseases"[Title/Abstract] OR "chronic kid-ney dis-ease"[Title/Abstract] OR "disease chronic kidney"[Title/Abstract] OR "diseases chronic kidney"[Title/Abstract] OR "kidney disease chronic"[Title/Abstract] OR "chronic renal diseases"[Title/Abstract] OR "chronic renal disease"[Title/Abstract] OR "disease chronic renal"[Title/Abstract] OR ("food safety"[MeSH Terms] OR ("Food"[All Fields] AND "Safe-ty"[All Fields]) OR "food safety"[All Fields]) OR "food safety"[Title/Abstract] OR "safety food"[Title/Abstract] OR "food hygiene"[Title/Abstract] OR "food*"[Title/Abstract] OR "nu-tritional well being"[Title/Abstract]) AND ("chronic kidney disease"[Title/Abstract] OR "renal insufficiency chronic"[Title/Abstract] OR "chronic renal insuffi-cien-cies"[Title/Abstract] OR (("renal insufficiency"[MeSH Terms] OR ("Renal"[All Fields] AND "Insufficiency"[All Fields]) OR "renal insufficiency"[All Fields] OR ("Renal"[All Fields] AND "Insufficiencies"[All Fields]) OR "renal insufficiencies"[All Fields]) AND "Chron-ic"[Title/Abstract]) OR "chronic renal insufficiency"[Title/Abstract] OR "kidney insuffi-ciency chronic"[Title/Abstract] OR "chronic kidney insufficiency"[Title/Abstract] OR (("Chronic"[All Fields] OR "chronical"[All Fields] OR "chronically"[All Fields] OR "chro-nicities"[All Fields] OR "chronicity"[All Fields] OR "chronicization"[All Fields] OR "chronics"[All Fields]) AND "kidney insufficiencies"[Title/Abstract]) OR (("renal insuf-fi-ciency"[MeSH Terms] OR ("Renal"[All Fields] AND "Insufficiency"[All Fields]) OR "re-nal insufficiency"[All Fields] OR ("Kidney"[All Fields] AND "Insufficiencies"[All Fields]) OR "kidney insufficiencies"[All Fields]) AND "Chronic"[Title/Abstract]) OR "chronic kid-ney diseases"[Title/Abstract] OR "chronic kidney disease"[Title/Abstract] OR "disease chronic kidney"[Title/Abstract] OR "diseases chronic kidney"[Title/Abstract] OR "kidney disease chronic"[Title/Abstract] OR "chronic renal diseases"[Title/Abstract] OR "chronic renal disease"[Title/Abstract] OR "disease chronic renal"[Title/Abstract])
